# Supplementary material for: ENO1 Promotes OSCC Migration and Invasion by Orchestrating IL-6 Secretion from Macrophages via a Positive Feedback Loop
Source: Int J Mol Sci. 2023 Jan 1;24(1):737. doi: 10.3390/ijms24010737 (PMC9821438; doi:10.3390/ijms24010737)
Supplement: Supplementary file 1 [file ijms-24-00737-s001.zip › Supplementary material-Figure S1 Original Images for Blots.pdf]

Figure S1. Original Images for Western Blot

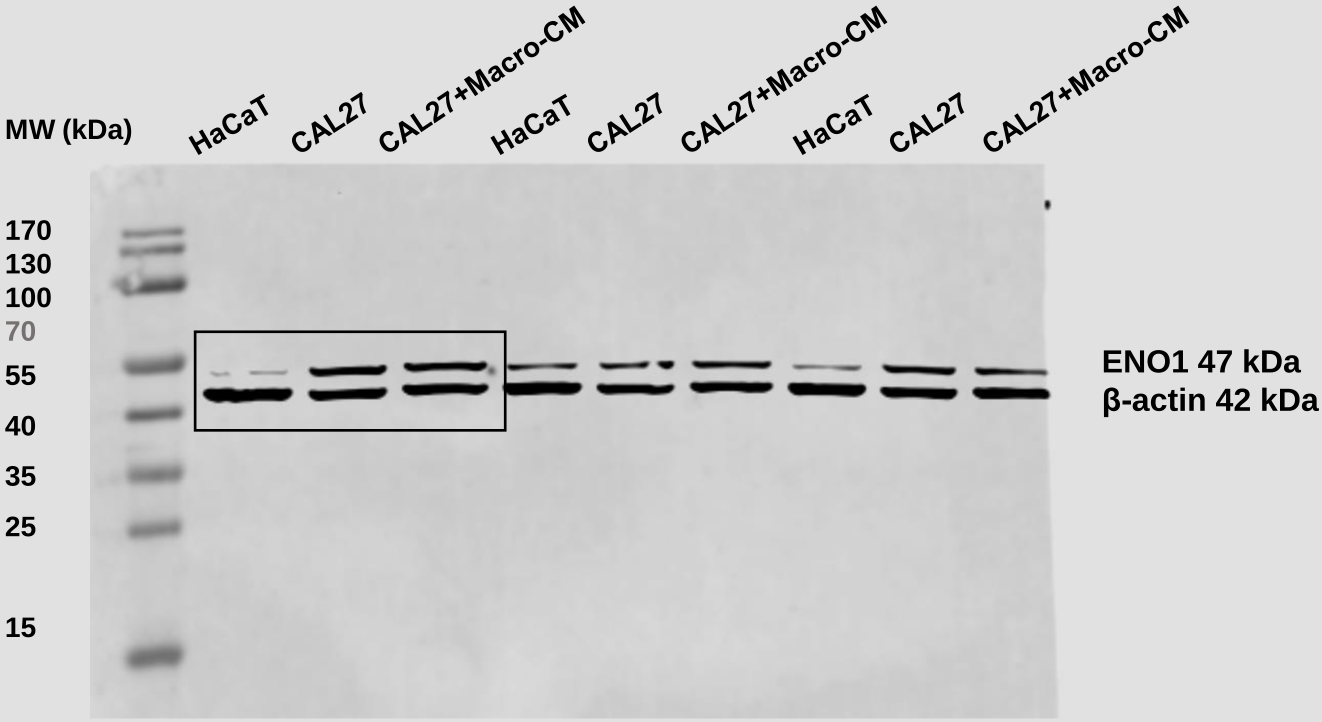

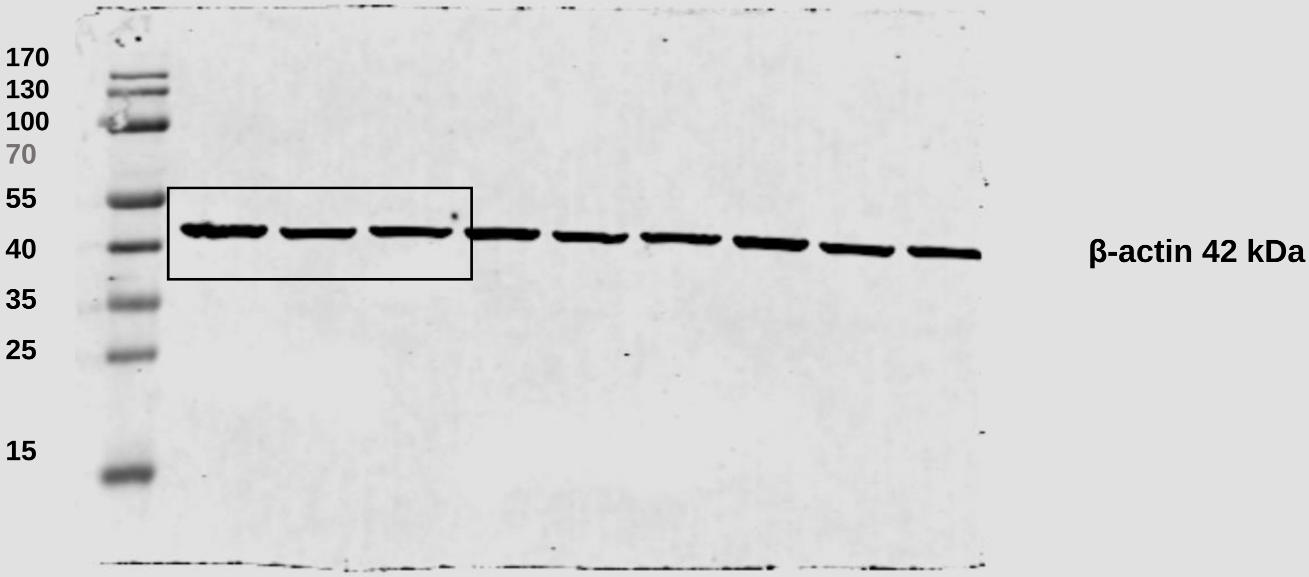

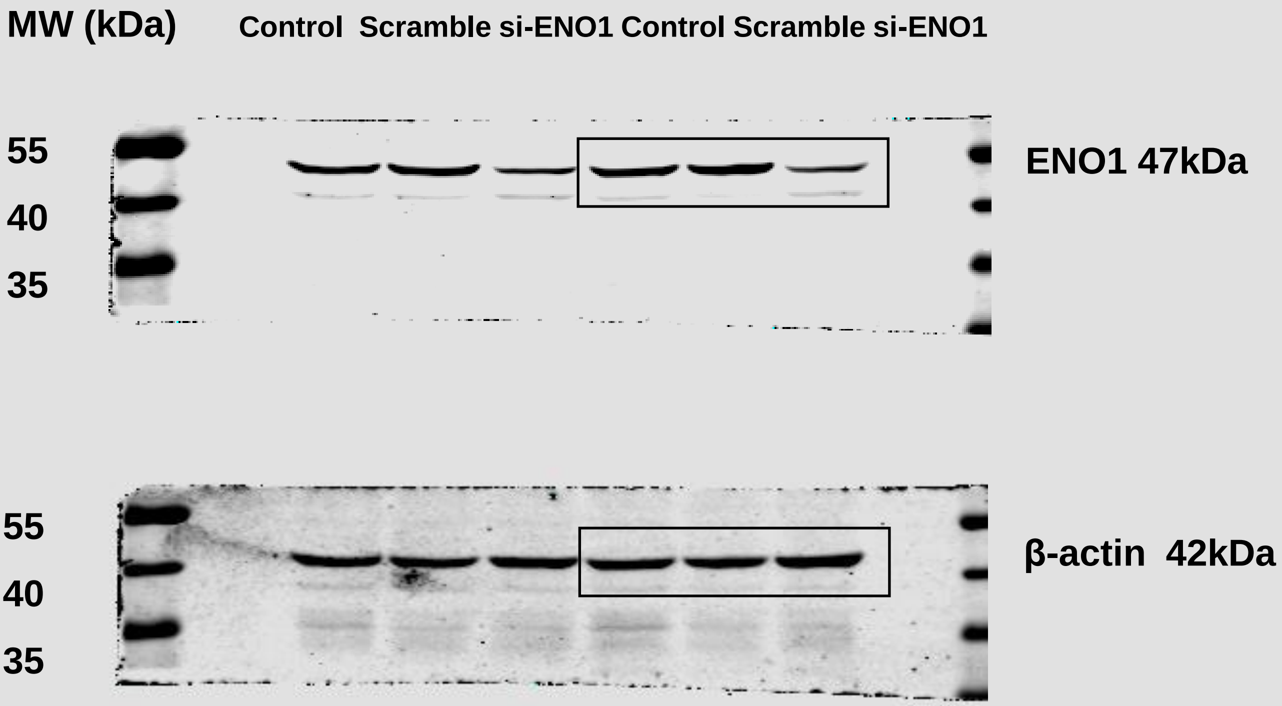
MW (kDa)
Control Scramble si-ENO1 Control Scramble si-ENO1
55
40
35
ENO1 47kDa
55
40
35
β-actin 42kDa

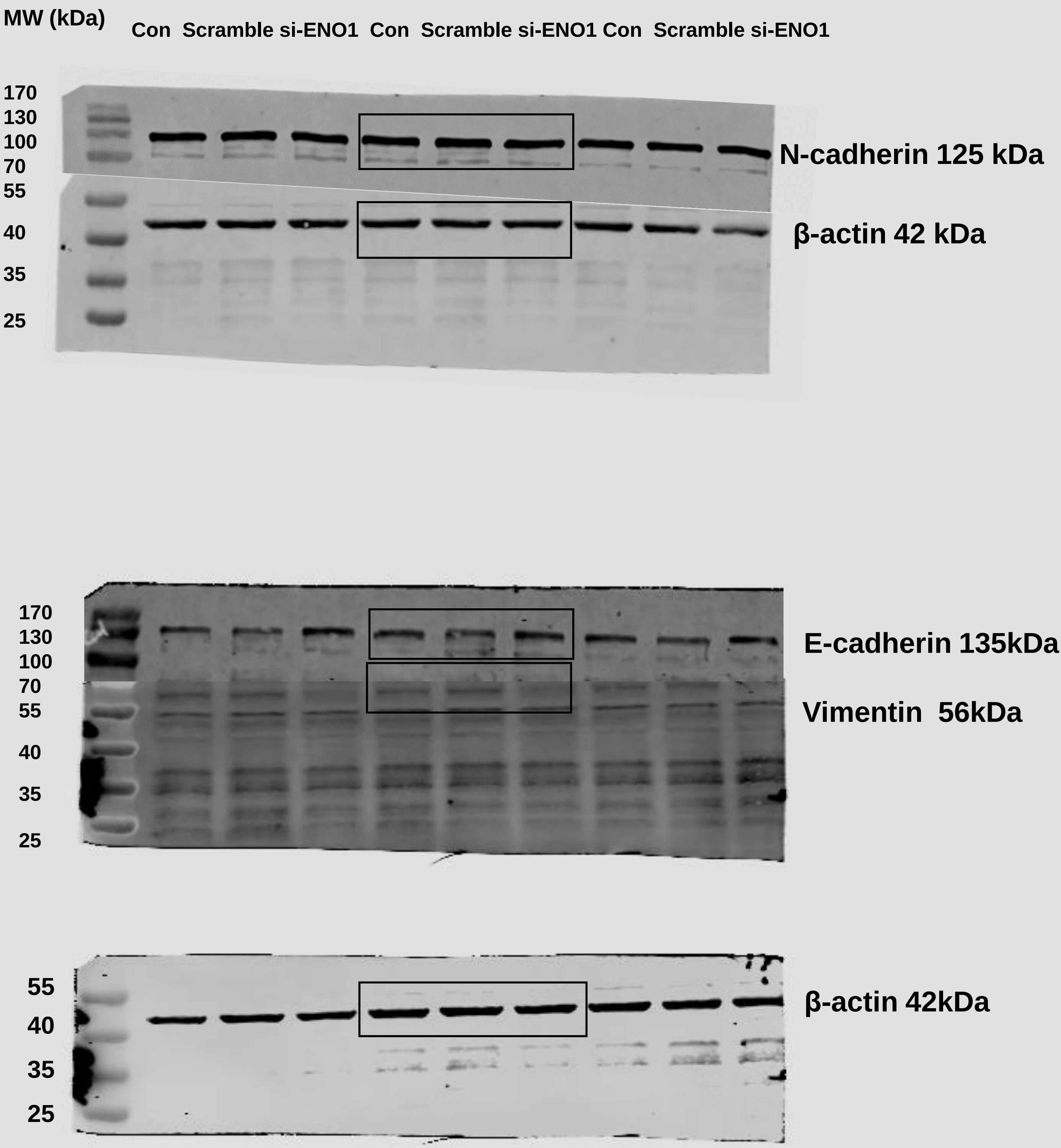
MW (kDa)
Con Scramble si-ENO1 Con Scramble si-ENO1 Con Scramble si-ENO1
170
130
100
70
55
40
35
25
N-cadherin 125 kDa
β-actin 42 kDa
170
130
100
70
55
40
35
25
E-cadherin 135kDa
Vimentin 56kDa
55
40
35
25
β-actin 42kDa

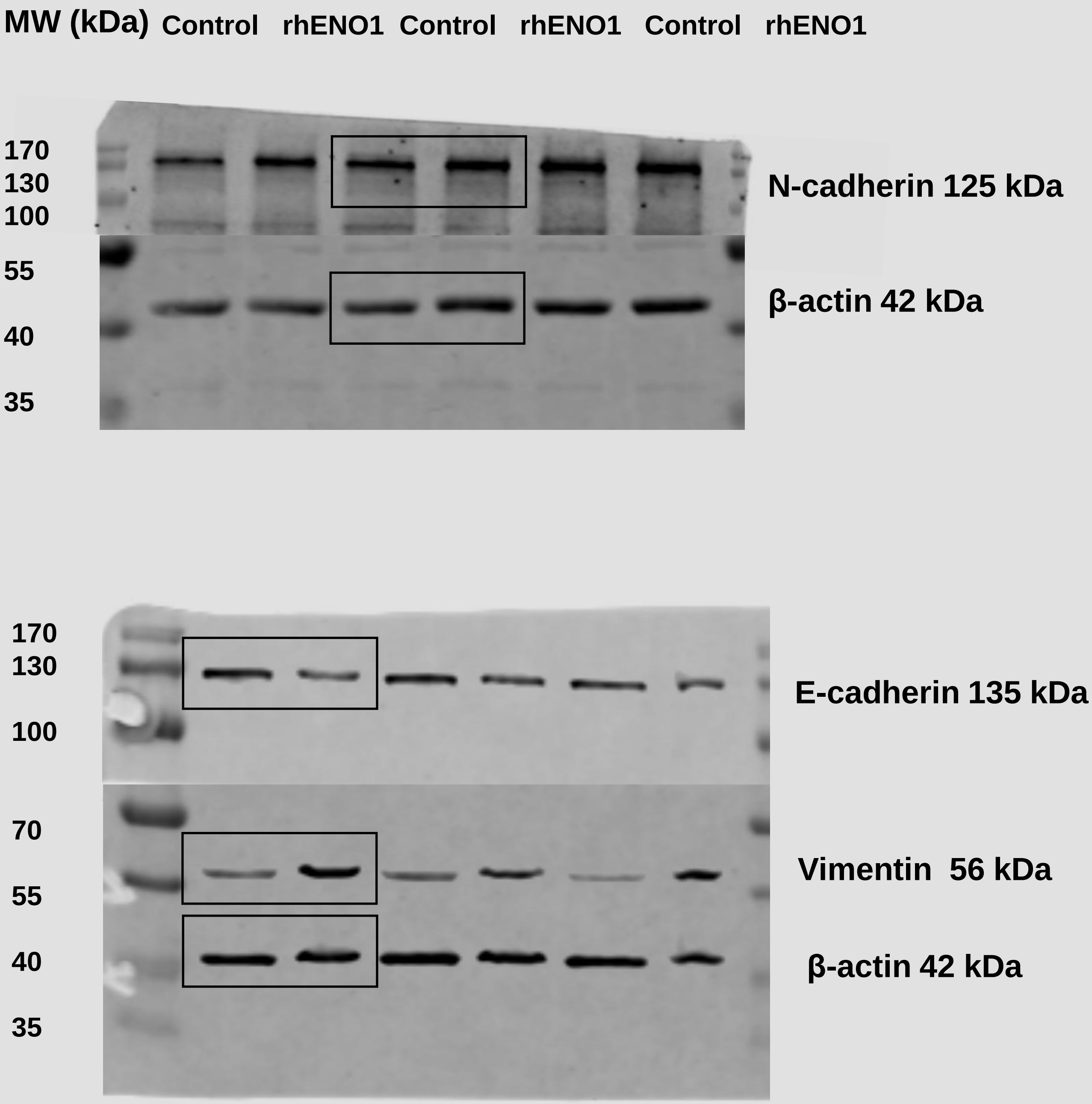
MW (kDa)
Control
rhENO1
Control
rhENO1
Control
rhENO1
170
130
100
55
40
35
N-cadherin 125 kDa
β-actin 42 kDa
170
130
100
70
55
40
35
E-cadherin 135 kDa
Vimentin 56 kDa
β-actin 42 kDa
