# Supplementary material for: ENO1 Promotes OSCC Migration and Invasion by Orchestrating IL-6 Secretion from Macrophages via a Positive Feedback Loop
Source: Int J Mol Sci. 2023 Jan 1;24(1):737. doi: 10.3390/ijms24010737 (PMC9821438; doi:10.3390/ijms24010737)
Supplement: Supplementary file 1 [file ijms-24-00737-s001.zip › Supplementary material-Table S1-2.pdf]

SUPPLEMENTARY TABLES

Table S1. Target sequences for siRNA

siRNA	Target sequence
NC siRNA	sense（5'-3'）：UUCUCCGAACGUGUCACGUTT<br>antisense（5'-3'）：ACGUGACACGUUCGGAGAATT
ENO1 siRNA	sense（5'-3'）：GCAUUGGAGCAGAGGUUUATT<br>antisense（5'-3'）：UAAACCUCUGCUCCAAUGCTT

Table S2. Primer sequences for RT-qPCR

Gene	Forward primer 5′-3′	Reverse primer 5′-3′
ENO1	GTACCGCCACATCGCTGACTTG	AGCATGAGAACCGCCATTGATGAC
ACTB	CCTGGCACCCAGCACAAT	GGGCCGGACTCGTCATAC
Actb	GTGCTATGTTGCTCTAGACTTCG	ATGCCACAGGATTCCATACC
Il6	ACTTCCATCCAGTTGCCTTCTTGG	TTAAGCCTCCGACTTGTGAAGTGG
Il10	CTGCTATGCTGCCTGCTCTTACTG	ATGTGGCTCTGGCCGACTGG
Il12b	CCTGTGACACGCCTGAAGAAGATG	CTTGTGGAGCAGCAGATGTGAGTG
Tnfa	CTCATGCACCACCATCAAGGACTC	AGACAGAGGCAACCTGACCACTC
Tgfb1	GCAACAATTCCTGGCGTTACCTTG	CAGCCACTGCCGTACAACTCC
